# High-Level Aminoglycoside Resistance in Human Clinical *Klebsiella pneumoniae* Complex Isolates and Characteristics of *armA*-Carrying IncHI5 Plasmids

**DOI:** 10.3389/fmicb.2021.636396

**Published:** 2021-04-07

**Authors:** Xueya Zhang, Qiaoling Li, Hailong Lin, Wangxiao Zhou, Changrui Qian, Zhewei Sun, Li Lin, Hongmao Liu, Junwan Lu, Xi Lin, Kewei Li, Teng Xu, Hailin Zhang, Changchong Li, Qiyu Bao

**Affiliations:** ^1^Department of Pediatric Respiratory Disease, The Second Affiliated Hospital and Yuying Children’s Hospital, Wenzhou Medical University, Wenzhou, China; ^2^Key Laboratory of Medical Genetics of Zhejiang Province, Key Laboratory of Laboratory Medicine, Ministry of Education of China, School of Laboratory Medicine and Life Sciences, Wenzhou Medical University, Wenzhou, China; ^3^Department of Laboratory Sciences, The Second Affiliated Hospital and Yuying Children’s Hospital, Wenzhou Medical University, Wenzhou, China; ^4^Institute of Translational Medicine, Baotou Central Hospital, Baotou, China; ^5^Institute of Biomedical Informatics, School of Laboratory Medicine and Life Sciences, Wenzhou Medical University, Wenzhou, China

**Keywords:** plasmid, IncHI5, *armA*, high-level aminoglycoside resistance, *Klebsiella pneumoniae*

## Abstract

Aminoglycosides are important options for treating life-threatening infections. However, high levels of aminoglycoside resistance (HLAR) among *Klebsiella pneumoniae* isolates have been observed to be increasing frequently. In this study, a total of 292 isolates of the *K. pneumoniae* complex from a teaching hospital in China were analyzed. Among these isolates, the percentage of HLAR strains was 13.7% (40/292), and 15 aminoglycoside resistance genes were identified among the HLAR strains, with *rmtB* being the most dominant resistance gene (70%, 28/40). We also described an *armA*-carrying *Klebsiella variicola* strain KP2757 that exhibited a high-level resistance to all aminoglycosides tested. Whole-genome sequencing of KP2757 demonstrated that the strain contained one chromosome and three plasmids, with all the aminoglycoside resistance genes (including two copies of *armA* and six AME genes) being located on a conjugative plasmid, p2757-346, belonging to type IncHI5. Comparative genomic analysis of eight IncHI5 plasmids showed that six of them carried two copies of the intact *armA* gene in the complete or truncated Tn*1548* transposon. To the best of our knowledge, for the first time, we observed that two copies of *armA* together with six AME genes coexisted on the same plasmid in a strain of *K. variicola* with HLAR. Comparative genomic analysis of eight *armA*-carrying IncHI5 plasmids isolated from humans and sediment was performed, suggesting the potential for dissemination of these plasmids among bacteria from different sources. These results demonstrated the necessity of monitoring the prevalence of IncHI5 plasmids to restrict their worldwide dissemination.

## Introduction

*Klebsiella pneumoniae* (*K. pneumoniae*) is one of the most common pathogens responsible for human infections. The emergence of highly antibiotic-resistant *K. pneumoniae* has become a major challenge facing clinical management and global public health (Kathryn et al., 2015).

Aminoglycosides are important options for treating life-threatening infections and are generally administered in combination with β-lactam agents (Becker and Cooper, 2013). However, increasing rates of aminoglycoside resistance in *K. pneumoniae* have been reported in recent years (Nasiri et al., 2018). Moreover, an increase in the aminoglycoside resistance rate in *Escherichia coli* isolates has been reported in France, leading to global concerns (Caméléna et al., 2020). Studies of aminoglycoside resistance in different species or populations have subsequently been conducted. A high level of aminoglycoside resistance in *Acinetobacter baumannii* and *Pseudomonas aeruginosa* isolated from a hospital in Brazil was observed (Ballaben et al., 2018), and an alarmingly high resistance to aminoglycosides has also been reported in diseased animals and clinical isolates of *Enterobacterales* from China (Fang-you et al., 2010; Yu-Ting et al., 2013).

The most common mechanism of resistance to aminoglycosides involves aminoglycoside-modifying enzymes (AMEs). These enzymes include acetyltransferases (AACs), nucleotidyltransferases (ANTs), and phosphotransferases (APHs), which differ in their ability to modify aminoglycosides (Miró et al., 2013). Other mechanisms contributing to aminoglycoside resistance involve the upregulation of efflux pumps (Poole, 2004) and reduced intake of antibiotics into the bacterial cell (Nakamatsu et al., 2007); however, the production of 16S rRNA methyltransferase (16S-RMTase) is another important mechanism mediating resistance to nearly all clinically available aminoglycosides. Different types of 16S-RMTase genes, including *rmtA*, *rmtB*, *rmtC*, *rmtD*, *rmtE*, *rmtF*, *rmtG*, *rmtH*, *armA*, and *npmA*, have been identified (Doi et al., 2016). Of these genes, *rmtB* and *armA* represent the most widespread 16S rRNA methylase genes (Caméléna et al., 2020). Genes encoding AMEs or methylases can be carried in integrons or transposons located in a variety of plasmids, which may facilitate the rapid spread of 16S-RMTase genes and AME genes among bacterial species (Nagasawa et al., 2014).

The H incompatibility (IncH) group is composed of IncHI and IncHII. IncHI plasmids can be further divided into five subgroups, IncHI1 to IncHI5, based on the different replication genes they contain (Liang et al., 2018). Currently, only 10 *armA*-carrying IncHI5 plasmids have been sequenced (last accessed on June 1st, 2020): pWLK-238,550 (CP038277), p13450-1 (CP026014), an unnamed plasmid (CP022441), pRo24724 (CP021328), pSIM-1-BJ01 (MH681289), p19051-IMP (MF344565), p13450-IMP (MF344564), p12208-IMP (MF344562), p11219-IMP (MF344561; Liang et al., 2018), and pKOX_R1 (CP003684; Huang et al., 2013). In this work, based on antimicrobial susceptibility testing, molecular biological methods, and whole-genome sequencing, the molecular resistance mechanisms of the clinical *K. pneumonia* complex, especially a high-level aminoglycoside-resistant strain that carries two copies of *armA* and an additional six AME genes, were analyzed.

## Materials and Methods

### Bacterial Strains

A total of 292 nonrepetitive isolates of the *K. pneumonia* complex were isolated from sputum, urine, blood, secretion, and pus samples for routine examination from 2015 to 2017 in an affiliated hospital of Wenzhou Medical University in Zhejiang Province, China. These strains were identified using a VITEK 2 automated microbiology analyzer (BioMerieux Corporate, Craponne, France). Given that the strains could not be distinguished into exact species on the basis of their biological phenotypes and multilocus sequence typing (MLST; Long et al., 2017; Yatao et al., 2018), we referred to them collectively as the *K. pneumonia* complex.

### Antimicrobial Susceptibility Testing

The minimum inhibitory concentrations (MICs) were determined by the agar dilution method and interpreted in accordance with the Clinical and Laboratory Standards Institute (CLSI) guidelines. As no breakpoint criteria for streptomycin were established by the European Committee on Antimicrobial Susceptibility Testing (EUCAST) or CLSI, the corresponding results were interpreted according to the criteria of the US Food and Drug Administration (Ya et al., 2017). High-level resistance of the bacterium to aminoglycosides was defined according to the MICs of certain antibiotics. For streptomycin (Pericàs et al., 2017) and kanamycin, MIC was ≥1,024 mg/L. For netilmicin, the MIC was ≥512 mg/L, while for gentamicin, amikacin, and tobramycin, the MIC was ≥256 mg/L (Caméléna et al., 2020). *E. coli* ATCC 25922 was used as a quality control strain.

### Genome Sequencing, Assembly, Annotation, and Bioinformatic Analysis

In order to identify aminoglycoside resistance genes that were carried by all strains, we performed pooled genomic DNA sequencing for 292 isolates. Each strain was cultured in 5 ml of lysogeny broth (LB) at 37°C for 16 h, and the strains were subsequently pooled together. Whole-cell DNA was extracted using an AxyPrep Bacterial Genomic DNA Miniprep Kit (Axygen Scientific, Union City, CA, United States) and pooled DNA sequencing was performed at Shanghai Sunny Biotechnology Co., Ltd. (Shanghai, China). Megahit (Li et al., 2015) was used to assemble the pooled sequencing data. In addition, three strains (KP2757, KP2723, and KP1878) showing high-level resistance to aminoglycosides were selected for whole-genome sequencing (WGS). Genomic DNA was extracted using the AxyPrep Bacterial Genomic DNA Miniprep kit and sequenced with Illumina NovaSeq 6,000 with 2 × 150-bp paired-end libraries (Illumina, Inc., San Diego, CA, United States) and PacBio RS II (Pacific Biosciences) systems with a 20-kb library at Shanghai Sunny Biotechnology Co., Ltd. (Shanghai, China). The Illumina reads were subjected to adaptor trimming and quality filtering (Phred quality score >20). Reads from PacBio RS II sequencing were assembled using the Canu v1.8 pipeline (Koren et al., 2017), and the quality of the genomic sequence was further corrected by reads from Illumina NovaSeq 6,000 sequencing.

Open reading frame (ORF) prediction of the pooled sequence data was performed using Prodigal, with the default parameters. Antibiotic resistance genes were predicted using both the Comprehensive Antibiotic Resistance Database (CARD; Moura et al., 2009) and the ResFinder (Jia et al., 2017) database. The relative abundance (sequencing depth) of a certain gene was calculated using BBMap.^1^ Potential ORFs of the whole genome of an isolate were predicted using the RAST pipeline (Overbeek et al., 2014) and annotated against the UniProt/SwissProt and nonredundant protein databases using the BLASTX program, with an e-value threshold of 1e-5. Annotation of mobile genetic elements and resistance genes was performed using ISfinder (Siguier et al., 2006), INTEGRALL, CARD, and ResFinder, with the default parameters being employed accordingly.

Basic genomic features were constructed using BLAST Ring Image Generator (BRIG; Nabil-Fareed et al., 2011). The comparison of nucleotide sequences was visualized using the genoPlotR package.^2^ A SNP tree was reconstructed with the pipeline CSI phylogeny accessible from the Center for Genomic Epidemiology.^3^

### Detection of Aminoglycoside Resistance Genes by PCR

The distribution of 16 aminoglycoside resistance genes was screened *via* PCR. The primers used for the resistance genes and their expected amplicon sizes and annealing temperatures are shown in Supplementary Table S1. The PCR products were sequenced, and the sequences were compared with those in the NCBI nucleotide database using BLAST.^4^

### Conjugation Experiments

Conjugation experiments were performed with sodium azide-resistant *E. coli* J53 as the recipient and the *armA*-positive isolate KP2757 as the donor. Briefly, a mixture of the donor and recipient strains was placed onto a sterile nitrocellulose filter, which was subsequently placed on a brain heart infusion (BHI) agar plate and incubated at 24°C for 24 h. The transconjugant was selected on a Muller-Hinton (MH) agar plate containing 256 mg/L sodium azide plus 64 mg/L amikacin. The existence of *armA* and additional AMEs in the transconjugant was verified by PCR and Sanger sequencing. Antimicrobial susceptibility testing was further performed for the transconjugant.

### Nucleotide Sequence Accession Numbers

The complete nucleotide sequences of chromosome KP2757 and plasmids p2757-138, p2757-40, and p2757-346 were submitted to GenBank under accession numbers CP060807–CP060810.

## Results

### Susceptibility Testing

MICs of six aminoglycosides against 292 *K. pneumonia* complex isolates showed that the resistance rates for gentamicin, kanamycin, tobramycin, netilmicin, amikacin, and streptomycin were 14.73% (43/292), 14.38% (42/292), 12.33% (36/292), 11.3% (33/292), 9.93% (29/292), and 9.59% (28/292), respectively. Although the resistance percentages for these six antibiotics did not exhibit notable differences (ranging from 9.59 to 14.73%), MICs, especially MIC_90_ values, differed considerably (>64-fold difference) among the antibiotics. MIC_90_ values for amikacin (16 mg/L) and streptomycin (32 mg/L) were considerably lower than those of gentamicin (>256 mg/L) and kanamycin (>1,024 mg/L), respectively (Table 1). Of the 292 KPC isolates, 62 were resistant to at least one of the aminoglycosides, and the percentage of high levels of aminoglycoside resistance (HLAR) was 13.7% (40/292). All 40 high-level aminoglycoside-resistant strains showed resistance to kanamycin, with 97.2% (39/40) showing MICs of >1,024 mg/L and the remaining strain showing a MIC of 128 mg/L. In addition, 70% (28/40) showed high MICs for five aminoglycosides (MICs of ≥1,024 mg/L for kanamycin; ≥256 mg/L for gentamicin, amikacin, and tobramycin; and ≥512 mg/L for netilmicin). Of note, KP2757 was the only strain showing high MICs for all six antibiotics (including MICs ≥1,024 mg/L for streptomycin). Ten strains showed a high MIC (>1,024 mg/L) for one antibiotic (kanamycin), while one strain (KP2723) showed a high MIC (256 mg/L) for gentamicin (Supplementary Table S2). The MICs of other classes of antimicrobials are shown in Supplementary Table S3.

**Table 1:** *In vitro* susceptibility to six aminoglycoside antibiotics for 292 isolates of the *Klebsiella pneumoniae* complex (mg/L).

Antibiotics	Breakpoints (CLSI)	Range	MIC_50_	MIC_90_	Resistance (%)
Amikacin	≥64	0.125–>1,024	1	16	9.93
Gentamicin	≥16	0.125–>256	0.25	>256	14.73
Kanamycin	≥64	0.25–>1,024	1	>1,024	14.38
Netilmicin	≥32	0.125–>512	0.25	128	11.3
Tobramycin	≥16	0.125–>256	0.25	>256	12.33
Streptomycin	≥64^a^	0.125–>1,024	2	32	9.59

MIC_50/90_, MIC that inhibits 50/90% of the isolates, respectively.

a The clinical breakpoint is defined by the US Food and Drug Administration.

### Detection of Aminoglycoside Resistance Genes

The pooled DNA sequencing of the 292 isolates predicted 16 aminoglycoside resistance genes, of which 15 were identified among the 40 HLAR strains [the most dominant resistance gene was *rmtB* (70%, 28/40)]. In addition, 55% (22/40) of the isolates carried two or more resistance genes. The most prevalent combination of the resistance genes was *rmtB* and *aadA2*, and 15% (6/40) of the strains had this combination of genes. Of the 29 16S-RMTase gene-positive strains, strain KP2757 carried the most aminoglycoside resistance genes, namely, *aac(3)-IId*, *aac(6´)-Ib3*, *aph(3´´)-Ib*, *aph(6)-Id*, *aadA2*, *aadA5*, and *armA* (free of *rmtB*), while the other 28 isolates were *rmtB* positive. Ten of 16S-RMTase gene-negative strains showed a high MIC (>1,024 mg/L) for kanamycin, which carried at least one *aac(3)-IId* or *aph(3´)-Ia* gene. The aminoglycoside resistance genes of the 40 high-level aminoglycoside-resistant strains are shown in Supplementary Table S2.

### Overview of the Whole-Genome Sequence of KP2757

**Figure 1:**
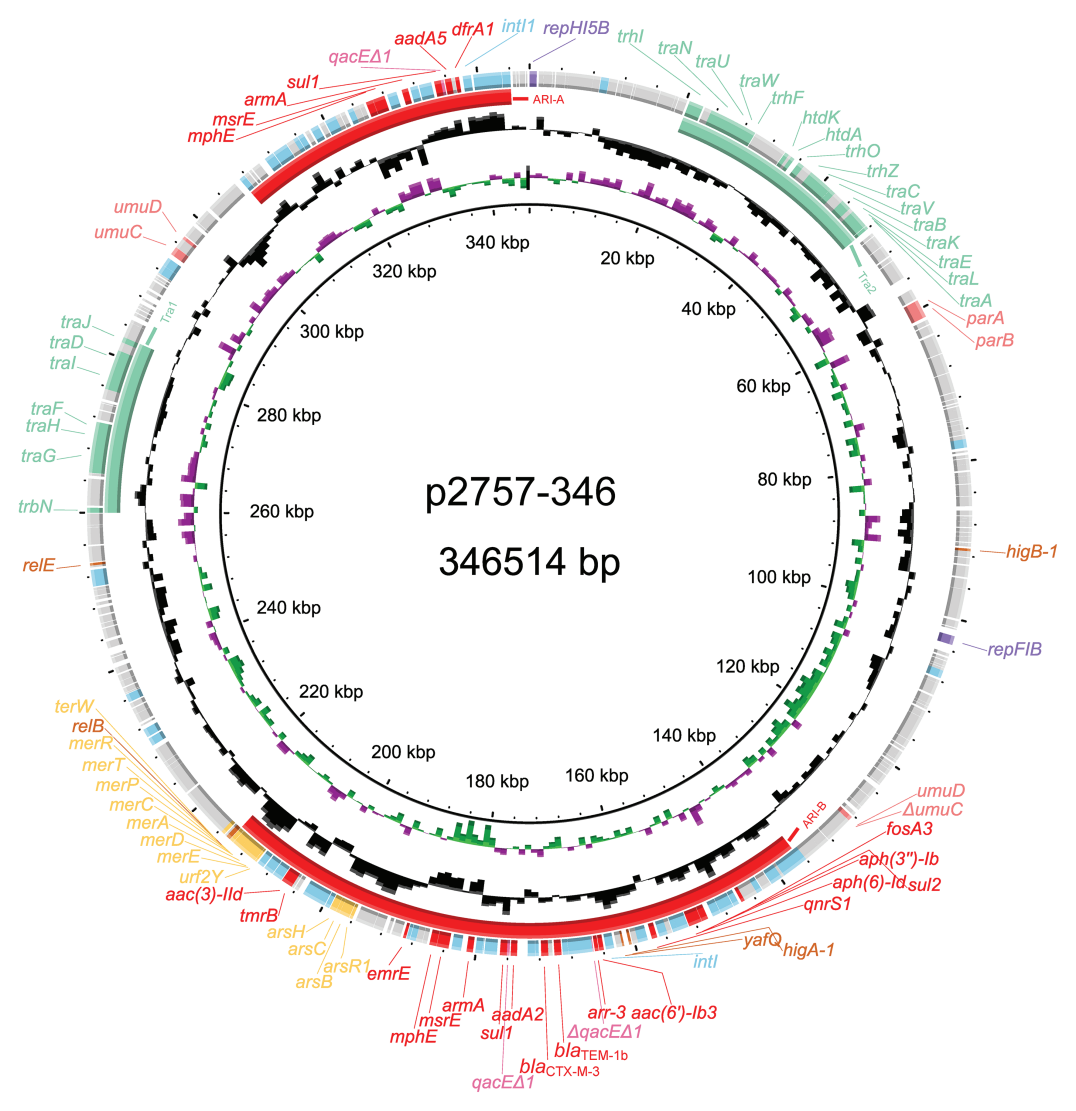
Schematic diagram of the p2757-346 plasmid. The various colors indicate different gene functions, including replication (in purple), transfer (in green), maintenance (in pink), and resistance (in red), and mobile elements (in blue). The circles show (from outside to inside) predicted coding sequences, the GC content, the GC skew, and the scale (in kilobases).

The complete genome sequences of three strains were obtained, and based on average nucleotide identity (ANI) analysis, KP2757 was identified as *Klebsiella variicola*, while the remaining strains were *K. pneumonia* strains. Annotation results of the genome sequences revealed the unique structure of the *armA*-related sequence in a plasmid of KP2757, so we focused on the analysis of KP2757 in this work. KP2757 contained a 5.21-Mb chromosome and three plasmids of different replicon types. Plasmid p2757-138 was an IncFII(K)/IncR plasmid, while p2757-40 was a nontypable plasmid according to PlasmidFinder analysis.^5^ Plasmid p2757-346 was 346,514 bp in size and was assigned to the IncHI5 group (Liang et al., 2018). This plasmid had an average G + C content of 47.25% and harbored 387 predicted ORFs. Sequence analysis demonstrated that p2757-346 contained a typical IncHI5 backbone (Liang et al., 2018), including genes involved in plasmid replication (*repHI5B* and *repFIB-like*), partitioning (*parA*, *parB*), and conjugative transfer (transfer regions *Tra1* and *Tra2*; Figure 1). Notably, in addition to genes providing resistance to β-lactams (*bla*_CTX-M-3_ and *bla*_TEM-1b_), fosfomycin (*fosA3*), macrolides (*mphE* and *msrE*), rifampicin (*arr-3*), quinolones (*qnrS1*), sulfonamides (*sul1* and *sul2*), and trimethoprim (*dfrA1*), p2757-346 carried eight aminoglycoside resistance genes [two copies of *armA*, *aac(3)-IId*, *aac(6´)-Ib3*, *aph(3´´)-Ib*, *aph(6)-Id*, *aadA2*, and *aadA5*]. Heavy metal resistance genes were also identified on plasmid p2757-346. Sequence analysis showed that plasmid p2757-346 carried different types of mobile genetic elements (MGEs), including a number of IS elements (e.g., IS*5075*, IS*26*, IS*903*, IS*Ec33*, IS*Ec28*, IS*Ec29*, IS*CR1*, IS*Vsa5*, IS*6100*, IS*Kpn28*, IS*Kpn26*, IS*Kpn21*, IS*Kpn19*, IS*4321R*, and IS*Bcen27*) and class 1 integrons (Figure 1).

### Comparative Genomic Analyses of the Backbone Sequences of *armA*-Carrying IncHI5 Plasmids

**Figure 2:**
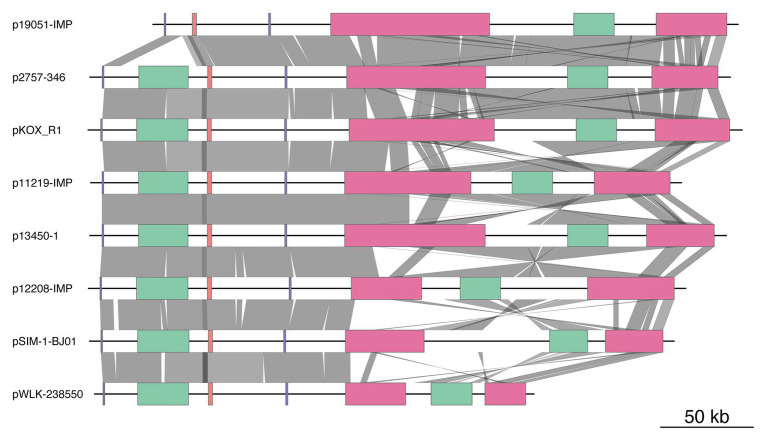
Comparison of the genome structure of eight plasmids. The color-coded boxes indicate different gene functions, including replication (in purple), plasmid transfer (in green), plasmid partition (in pink), and antibiotic resistance island (ARI; in red). Orthologous regions are connected and color coded. The plasmids and their accession numbers are p2757-346 (CP060810), pWLK-238,550 (CP038277), p13450-1 (CP026014), pSIM-1-BJ01 (MH681289), p19051-IMP (MF344565), p12208-IMP (MF344562), p11219-IMP (MF344561), and pKOX_R1 (CP003684). The shading indicates 90% nucleotide sequence identity.

By searching the NCBI nucleotide database and using sequences of the combination of replicon genes of IncHI5 plasmids and the *armA* gene as queries, we obtained the sequences of 10 *armA*-carrying IncHI5 plasmids. These plasmids were detected in *K. pneumoniae* [p11219-IMP, p12208-IMP, p13450-IMP, pSIM-1-BJ01, p19051-IMP, and unnamed plasmid (CP022441)], *K. variicola* (p13450-1), *Klebsiella michiganensis* (pKOX-R1), and *Raoultella ornithinolytica* (pWLK-238,550 and pRo24724). Sequences of all plasmids were complete, except for pRo24724. Sequence alignment revealed that two pairs of plasmids [pSIM-1-BJ01/unnamed plasmid (CP022441) and p13450-IMP/p13450-1] shared identical sequences. Therefore, three plasmids [pRo24724, unnamed plasmid (CP022441), and p13450-IMP] were omitted, and eight plasmid sequences were used for the comparative genomic analysis (Figure 2 and Supplementary Table S4).

The major IncHI5 backbone genes for replication, partition, and conjugal transfer were conserved among these eight plasmids. Two conjugal transfer regions, Tra1 and Tra2, were found in seven IncHI5 plasmids, but p19051-IMP had only a Tra1 region. Plasmid p2757-346 contained more than 20 conjugal transfer genes, including *traA*, *traB*, *traC*, *traD*, *traE*, *traF*, *trhF*, *htdK*, and *htdA*. Comparative genomic analysis indicated that the transfer regions of the four plasmids, pKOX-R1, p13450-1, p12208-IMP, and p11219-IMP, showed high similarity to that of p2757-346 (>95% coverage and >99% identity).

**Figure 3:**
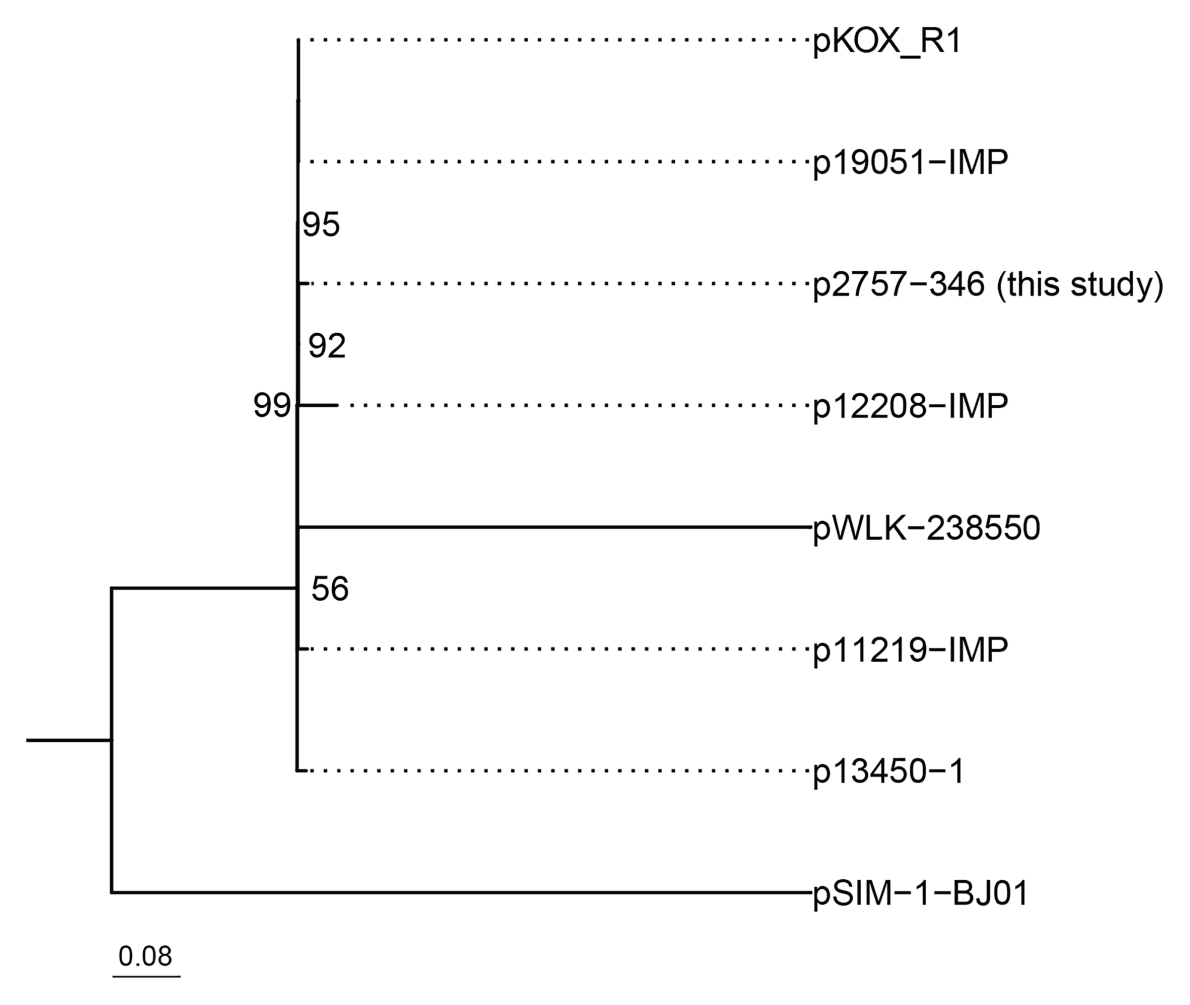
Phylogenetic tree of eight IncH5 plasmids. The bootstrap values are given at the nodes of the tree; values <50% are not shown. The bar corresponds to the scale of sequence divergence.

Phylogenetic analysis of these eight IncHI5 plasmids indicated that p2757-346 was closely related to plasmid pKOX-R1, the first sequenced IncHI5 plasmid, isolated from *K. michiganensis* (Huang et al., 2013) and plasmid p19051-IMP from *K. pneumoniae* (Liang et al., 2018; Figure 3).

### Comparative Genomic Analysis of Antibiotic Resistance Island

**Figure 4:**
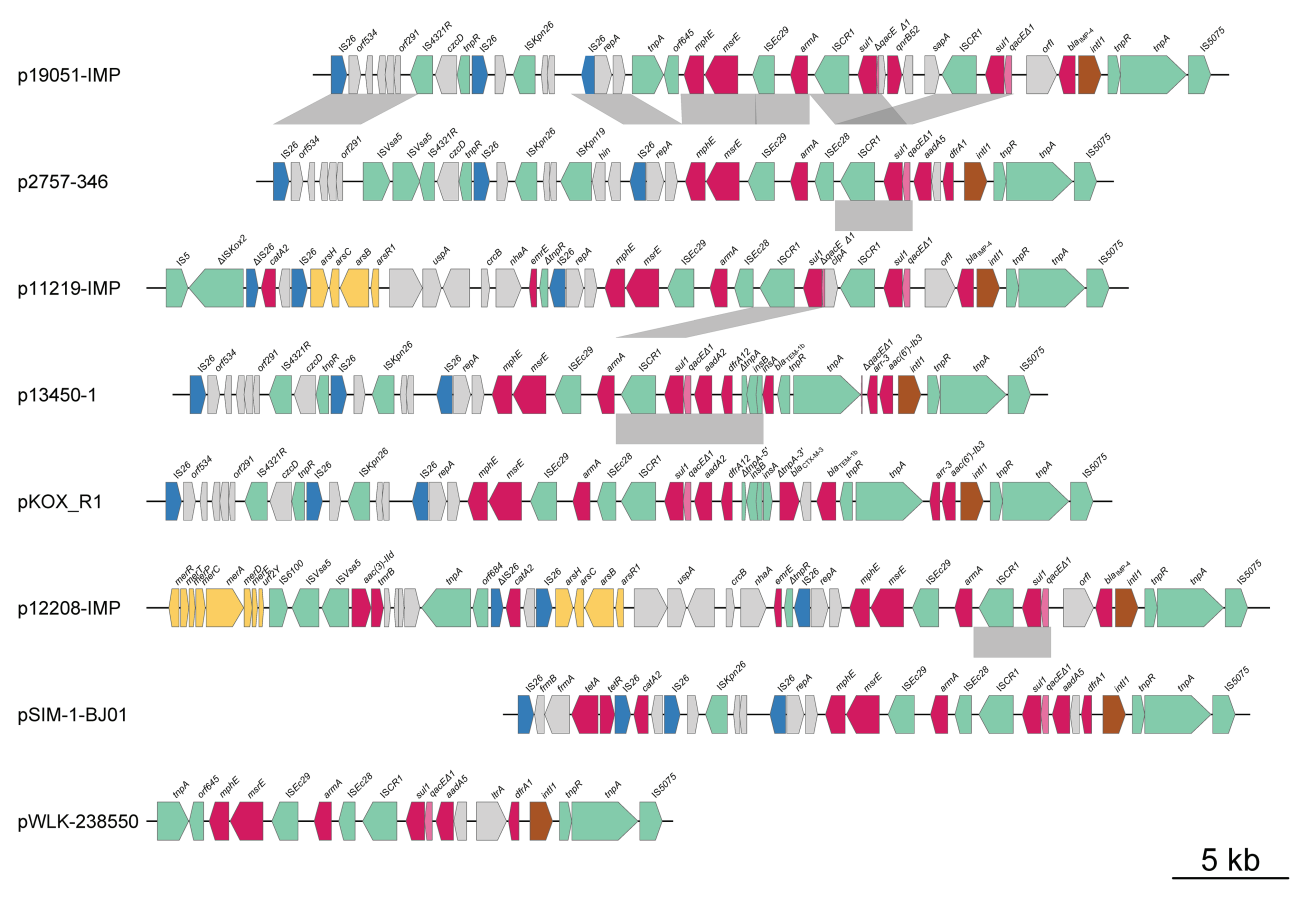
Comparative genomic analysis of antibiotic resistance island A (ARI-A). Mobile elements are shown by green, brown, and blue arrows. The red arrows indicate antibiotic resistance genes, the pink arrows denote biocide resistance genes, and the gray arrows represent genes of other functions. The shading indicates 100% nucleotide sequence identity.

**Figure 5:**
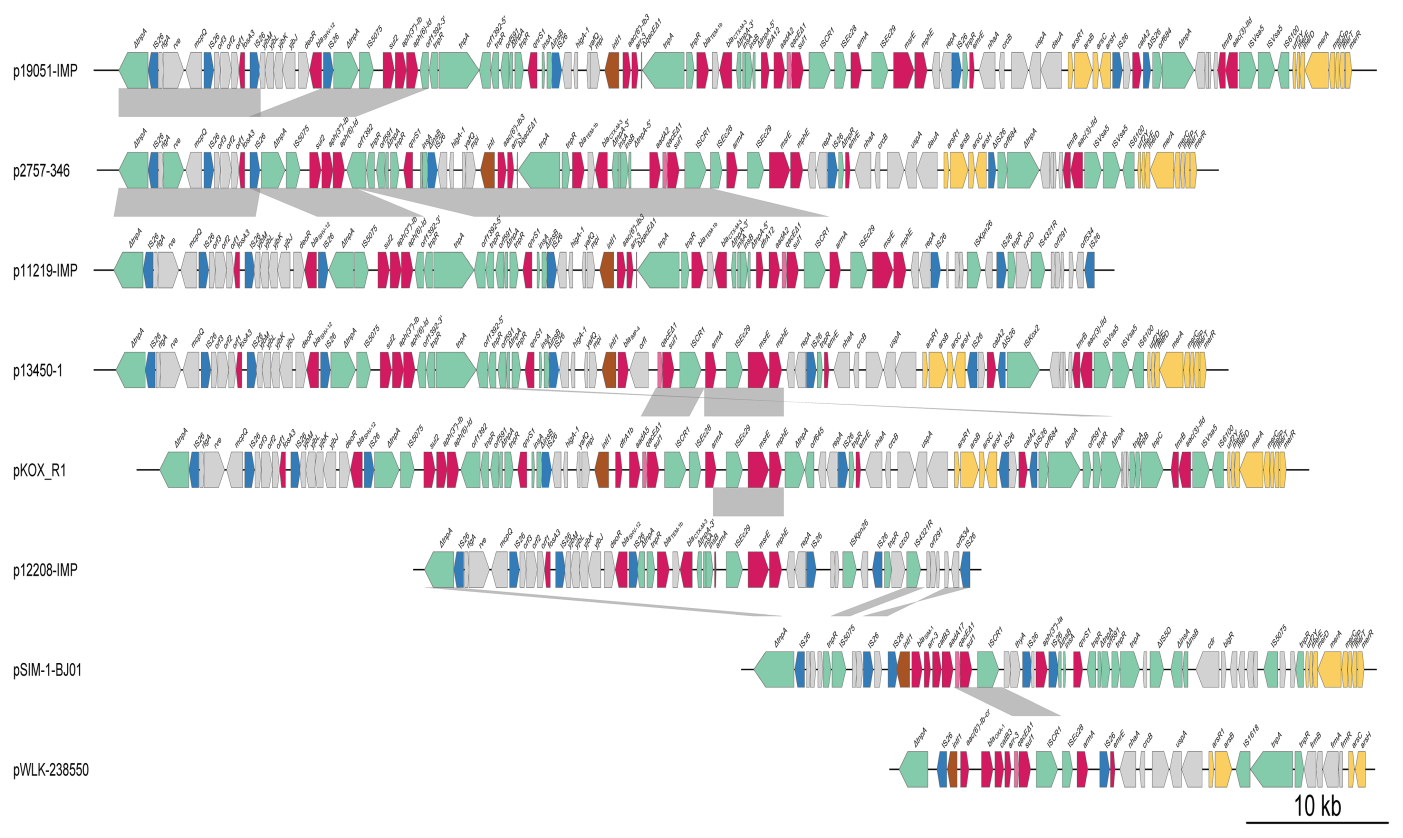
Comparative genomic analysis of antibiotic resistance island B (ARI-B). Mobile elements are shown by green, brown, and blue arrows, respectively. The red arrows indicate antibiotic resistance genes, the pink arrows denote biocide resistance genes, and the gray arrows represent genes of other functions. The shading indicates 100% nucleotide sequence identity.

Plasmid p2757-346 contained two antibiotic resistance islands (ARIs), designated ARI-A and ARI-B (Figures 1, 4, 5). ARI-A measured 36,950 bp in length and was bracketed by IS*26* and IS*5075* elements from positions 307,130 to 344,080, in which six resistance genes (*dfrA1*, *aadA5*, *sul1*, *armA*, *msrE*, and *mphE*) were encoded. ARI-B was 77,593 bp in size and started from the truncated transposase gene *ΔtnpA* of IS*EC63* at nucleotide sequence position 136,625 and ended in *merR* at 214,218. ARI-B encoded resistance genes providing resistance against various classes of antibiotics (aminoglycosides, β-lactams, fosfomycin, macrolides, rifampicin, quinolones, and sulfonamides), *mer* (*merRTPCADEY*) genes, and *ars* (*arsR1BCH*) resistance genes (Figure 1). Pairwise sequence comparisons *via* BLASTN showed that significant differences between the eight plasmids were observed in the ARIs. In addition, ARI-B in p19051-IMP had high similarity with that in p2757-346 (approximately 89% coverage and 99% identity).

All eight plasmids carried two copies of the intact *armA* gene (one encoded in ARI-A and the other in ARI-B), with the exceptions of pSIM-1-BJ01, which did not have an *armA* gene in the ARI-B region, and p12208-IMP, which had a truncated *armA* in the ARI-B region. Among these plasmids, the *armA* gene was located in the Tn1548 or truncated Tn1548 transposon (Figures 4, 5).

Both the ARI-A and ARI-B regions contained class 1 integrons, which carried genes that provided resistance to aminoglycosides [*aadA2*, *aadA5*, and *aac(6)-Ib3*], rifampicin (*arr-3*), trimethoprim (*dfr1*), and sulfonamides (*sul1*). The class 1 integron of ARI-B carried *bla*_TEM-1b_ and *bla*_CTX-M-3_. In addition, *sul2*, *aph(3´´)-Ib*, and *aph(6)-Id* were flanked by *tnpR* and IS*5075*, and this fragment was found in p19051-IMP, p11219-IMP, p13450-1, and pKOR-R1. The *aac(3)-IId* gene may have been mobilized by IS*Vsa5* in p2757-346, p19051-IMP, p13450-1, p12208-IMP, and pKOR-R1. The *fosA3* gene was associated with IS*26*, which was also found in p19051-IMP, p11219-IMP, p13450-1, pKOR-R1, and p12208-IMP. The distributions of aminoglycoside resistance genes in all plasmids are shown in Supplementary Table S4.

### Dissemination of *armA*-Carrying IncHI5 Plasmids

p2757-346 is a conjugative plasmid. The antibiotic susceptibilities of the transconjugant are shown in Table 2. Moreover, bacteria carrying the *armA*-encoding IncHI5 plasmids were isolated from humans and sediment (no data about bacteria of animal origin available), suggesting the potential of this plasmid in mediating the dissemination of the 16S-RMTase-encoding genes among humans and throughout the environment (Supplementary Table S4).

**Table 2:** Antibiotic susceptibilities of KP2757, the transconjugant, the recipient control *Escherichia coli* J53, and the quality control strain *E. coli* ATCC 25922 (mg/L).

Antibiotic	KP2757	p2757-346/*E. coli* J53	*E. coli* J53	*E. coli* ATCC 25922
Amikacin	>1,024	>1,024	0.5	2
Gentamicin	>256	>256	0.25	0.5
Kanamycin	>1,024	>1,024	1	4
Netilmicin	>512	>512	0.25	0.5
Tobramycin	>256	>256	0.25	0.5
Streptomycin	1,024	256	2	4

## Discussion

In this work, 13.7% (40/292) of clinical *K. pneumoniae* complex isolates collected in the period of 2015–2017 were high-level aminoglycoside-resistant strains. Most of them showed high MICs for aminoglycosides, especially kanamycin, with 97.2% (39/40) showing an MIC of >1,024 mg/L, and 9.9% (29/292) of strains showed high MICs for gentamicin, amikacin, and tobramycin. A previous study conducted in the same district (Wenzhou, China) found that only 6.2% (21/337) of the *K. pneumoniae* isolates collected from 2006 to 2007 showed high MICs to gentamicin, amikacin, and tobramycin (MIC ≥ 256 mg/L; Fangyou et al., 2009). The number of *K. pneumoniae* complex isolates resistant to all three antibiotics increased by approximately 3.7% (a relative increase of 59.7%) during the past 10 years. High-level aminoglycoside-resistant *K. pneumoniae* isolates have been reported in different districts and countries (Sabtcheva et al., 2008; Qiong et al., 2009). However, to the best of our knowledge, no other publication has reported the percentage of *K. pneumoniae* with HLAR, although high levels of aminoglycoside resistance in other species have been reported. The percentages of HLAR (MICs for both gentamicin and amikacin were higher than 512 mg/L) of the clinical *A. baumannii* isolates from Beijing, China, in 2006, 2007, 2008, and 2009 were 52.63, 65.22, 51.11, and 70.83%, respectively (Lu et al., 2014). Thirty of the 137 aminoglycoside-resistant *E. coli* collected from nine hospitals in France showed high-level resistance (MIC ≥ 256 mg/L) to gentamicin, amikacin, and tobramycin (Caméléna et al., 2020). The prevalence of high-level aminoglycoside-resistant bacteria is worrisome.

16S-RMTase genes (*rmtB* and *armA*) were identified among 72.5% (29/40) of high-level aminoglycoside-resistant strains in this study, of which *rmtB* (70%, 28/40) was much more prevalent than *armA* (2.5%, 1/40). These findings are in accordance with the observations of other studies in China, which were conducted in Wenzhou and Shanghai by other researchers (Fangyou et al., 2009; Qiong et al., 2009). Ten 16S-RMTase gene-negative strains showing a high MIC (>1,024 mg/L) for kanamycin carried at least one *aac(3)-IId* or *aph(3´)-Ia* gene, indicating that *aac(3)-IId* and *aph(3´)-Ia* may provide high-level resistance against kanamycin. Similarly, the production of 16S-RMTase or a combination of multiple AMEs was shown to be the major mechanism underlying high-level aminoglycoside-resistant gram-negative bacilli in Chengdu, China, and Brazil (Wang et al., 2016; Ballaben et al., 2018).

In this work, two copies of *armA* and multiple AME genes were found in a transferable IncHI5 plasmid. *armA* genes have previously been identified in IncL/M, IncA/C, IncX, IncHI1, and IncF plasmids (Dolejska et al., 2013; Caméléna et al., 2020). Compared with *armA*-positive IncHI5 plasmids, to the best of our knowledge, IncL/M, IncA/C, IncX, IncHI1, and IncF plasmids have never been reported to carry two copies of *armA*.

Comparative genomic analysis revealed that the genetic context of the *armA* gene was similar in IncHI5 plasmids from *K. pneumoniae*, *K. variicola*, and *K. michiganensis*. The module of *armA*-related transposable elements was highly similar to those of *armA* harboring IncFII plasmids (Xiang-Dang et al., 2012) and IncA/C plasmids (Patrick et al., 2012), suggesting that the *armA* module could disseminate among plasmids of different incompatibility groups.

Except for *armA* and multiple AME genes, extended-spectrum β-lactamase (ESBL) genes were colocalized with genes conferring resistance to fosfomycin, macrolides, rifampicin, quinolones, and sulfonamides on the IncHI5 plasmid p2757-346. Acquired 16S-RMTase genes were mainly identified in transferable plasmids and were always associated with ESBL and carbapenemase genes or other resistance genes (Laurent et al., 2014). Plasmids carrying resistance genes may cause the global spread of resistance determinants, constituting an emerging global threat. In recent years, in addition to the phenomenon of resistance genes of various antibiotic classes being identified in a single bacterium, bacteria with multiple copies of identical resistance genes or gene arrays have been detected more often (Naas et al., 2001; Ding-Qiang et al., 2016; Teng et al., 2018). This phenomenon makes the treatment of infectious diseases more difficult and threatens human health.

## Conclusion

To the best of our knowledge, we first described that two copies of *armA* together with six AME genes coexisted on one plasmid in a HLAR *K. variicola* isolate. Comparative genomic analysis showed that most *armA*-positive IncHI5 plasmids harbored two copies of the *armA* gene, which had not been previously reported in IncL/M, IncA/C, IncX, IncHI1, and IncF plasmids. Eight *armA*-encoding IncHI5 plasmids were isolated from humans and sediment, suggesting the potential for dissemination of this *armA*-carrying plasmid among bacteria of different sources. This result demonstrates the necessity of monitoring the prevalence of IncHI5 plasmids to restrict their worldwide dissemination.

## Data Availability Statement

The datasets presented in this study can be found in online repositories. The names of the repository/repositories and accession number(s) can be found in the article/Supplementary Material.

## Author Contributions

HZ, CL, and QB designed the study. XZ, QL, HaL, LL, HoL, JL, and XL acquired the data. WZ, CQ, and ZS performed the results analysis and interpreted the data, and XZ wrote the first draft of the paper. KL, TX, and QB revised the draft critically for important intellectual content. All authors contributed to the article and approved the submitted version.

### Conflict of Interest

The authors declare that the research was conducted in the absence of any commercial or financial relationships that could be construed as a potential conflict of interest.

## Footnotes

^1^http://sourceforge.net/projects/bbmap/

^2^http://genoplotr.r-forge.r-project.org

^3^www.genomicepidemiology.org

^4^http://www.ncbi.nlm.nih.gov/BLAST

^5^https://cge.cbs.dtu.dk/services/PlasmidFinder/

## References

[ref1] Ballaben A. S. Andrade L. N. Galetti R. Ferreira J. C. McElheny C. L. Mettus R. T.. (2018). Diversity of high-level aminoglycoside resistance mechanisms among gram-negative nosocomial pathogens in Brazil. Antimicrob. Agents Chemother. 62, e01550–e01618. 10.1128/AAC.01550-18, PMID: 30150471 PMC6201109

[ref2] Becker B. Cooper M. A. (2013). Aminoglycoside antibiotics in the 21st century. ACS Chem. Biol. 8, 105–115. 10.1021/cb3005116, PMID: 23110460

[ref3] Caméléna F. Morel F. Merimèche M. Decousser J. W. Jacquier H. Clermont O.. (2020). Genomic characterization of 16S rRNA methyltransferase-producing *Escherichia coli* isolates from the Parisian area, France. J. Antimicrob. Chemother. 75, 1726–1735. 10.1093/jac/dkaa105, PMID: 32300786

[ref4] Ding-Qiang C. Ai-Wu W. Ling Y. Dan-Hong S. Yong-Ping L. Yan-Wei H.. (2016). Emergence and plasmid analysis of *Klebsiella pneumoniae* KP01 carrying blaGES-5 from Guangzhou, China. Antimicrob. Agents Chemother. 60, 6362–6364. 10.1128/AAC.00764-16, PMID: 27431225 PMC5038298

[ref5] Doi Y. Wachino J. I. Arakawa Y. (2016). Aminoglycoside resistance: the emergence of acquired 16S ribosomal RNA methyltransferases. Infect. Dis. Clin. N. Am. 30, 523–537. 10.1016/j.idc.2016.02.011, PMID: 27208771 PMC4878400

[ref6] Dolejska M. Villa L. Poirel L. Nordmann P. Carattoli A. (2013). Complete sequencing of an IncHI1 plasmid encoding the carbapenemase NDM-1, the ArmA 16S RNA methylase and a resistance-nodulation-cell division/multidrug efflux pump. J. Antimicrob. Chemother. 68, 34–39. 10.1093/jac/dks357, PMID: 22969080

[ref7] Fang-you Y. Dan Y. Jing-ye P. Chong C. Zhi-qiang Q. Chris P.. (2010). High prevalence of plasmid-mediated 16S rRNA methylase gene rmtB among *Escherichia coli* clinical isolates from a Chinese teaching hospital. BMC Infect. Dis. 10:184. 10.1186/1471-2334-10-184, PMID: 20573216 PMC2905422

[ref8] Fangyou Y. Liangxing W. Jingye P. Dan Y. Chan C. Tao Z.. (2009). Prevalence of 16S rRNA methylase genes in *Klebsiella pneumoniae* isolates from a Chinese teaching hospital: coexistence of rmtB and armA genes in the same isolate. Diagn. Microbiol. Infect. Dis. 64, 57–63. 10.1016/j.diagmicrobio.2009.01.020, PMID: 19232867

[ref9] Huang T. W. Wang J. T. Lauderdale T. L. Liao T. L. Lai J. F. Tan M. C.. (2013). Complete sequences of two plasmids in a blaNDM-1-positive *Klebsiella* oxytoca isolate from Taiwan. Antimicrob. Agents Chemother. 57, 4072–4076. 10.1128/AAC.02266-12, PMID: 23752513 PMC3719732

[ref10] Jia B. Raphenya A. R. Alcock B. Waglechner N. Guo P. Tsang K. K.. (2017). CARD 2017: expansion and model-centric curation of the comprehensive antibiotic resistance database. Nucleic Acids Res. 45, D566–D573. 10.1093/nar/gkw1004, PMID: 27789705 PMC5210516

[ref11] Kathryn E. H. Heiman W. Ruth N. Z. Stephen B. Chris A. W. David D.. (2015). Genomic analysis of diversity, population structure, virulence, and antimicrobial resistance in *Klebsiella pneumoniae*, an urgent threat to public health. Proc. Natl. Acad. Sci. U. S. A. 112, E3574–E3581. 10.1073/pnas.1501049112, PMID: 26100894 PMC4500264

[ref12] Koren S. Walenz B. P. Berlin K. Miller J. R. Bergman N. H. Phillippy A. M. (2017). Canu: scalable and accurate long-read assembly via adaptive k-mer weighting and repeat separation. Genome Res. 27, 722–736. 10.1101/gr.215087.116, PMID: 28298431 PMC5411767

[ref13] Laurent P. Jaime L. Helia B. Maria Luisa R. Sandrine B. Patrice N. (2014). Emergence of the 16S rRNA methylase RmtG in an extended-spectrum-β-lactamase-producing and colistin-resistant *Klebsiella pneumoniae* isolate in Chile. Antimicrob. Agents Chemother. 58, 618–619. 10.1128/AAC.02059-13, PMID: 24165178 PMC3910725

[ref14] Li D. Liu C. M. Luo R. Sadakane K. Lam T. W. (2015). MEGAHIT: an ultra-fast single-node solution for large and complex metagenomics assembly via succinct de Bruijn graph. Bioinformatics 31, 1674–1676. 10.1093/bioinformatics/btv033, PMID: 25609793

[ref15] Liang Q. Jiang X. Hu L. Yin Z. Gao B. Zhao Y.. (2018). Sequencing and genomic diversity analysis of IncHI5 plasmids. Front. Microbiol. 9:3318. 10.3389/fmicb.2018.03318, PMID: 30692976 PMC6339943

[ref16] Long S. W. Sarah E. L. Matthew Ojeda S. Concepcion C. James J. D. Thomas B.. (2017). Whole-genome sequencing of human clinical *Klebsiella pneumoniae* isolates reveals misidentification and misunderstandings of *Klebsiella pneumoniae*, *Klebsiella variicola*, and *Klebsiella quasipneumoniae*. mSphere 2, e00290–e00317. 10.1128/mSphereDirect.00290-17, PMID: 28776045 PMC5541162

[ref17] Lu N. Yuemeng L. Min Y. Xinxin H. Tongying N. Xinyi Y.. (2014). Genetic basis of high level aminoglycoside resistance in *Acinetobacter baumannii* from Beijing, China. Acta Pharm. Sin. B 4, 295–300. 10.1016/j.apsb.2014.06.004, PMID: 26579398 PMC4629078

[ref18] Miró E. Grünbaum F. Gómez L. Rivera A. Mirelis B. Coll P.. (2013). Characterization of aminoglycoside-modifying enzymes in enterobacteriaceae clinical strains and characterization of the plasmids implicated in their diffusion. Microb. Drug Resist. 19, 94–99. 10.1089/mdr.2012.0125, PMID: 23206280

[ref19] Moura A. Soares M. Pereira C. Leitão N. Henriques I. Correia A. (2009). INTEGRALL: a database and search engine for integrons, integrases and gene cassettes. Bioinformatics 25, 1096–1098. 10.1093/bioinformatics/btp105, PMID: 19228805

[ref20] Naas T. Mikami Y. Imai T. Poirel L. Nordmann P. (2001). Characterization of In53, a class 1 plasmid- and composite transposon-located integron of *Escherichia coli* which carries an unusual array of gene cassettes. J. Bacteriol. 183, 235–249. 10.1128/JB.183.1.235-249.2001, PMID: 11114922 PMC94871

[ref21] Nabil-Fareed A. Nicola K. P. Nouri L. B. Z. Scott A. B. (2011). BLAST ring image generator (BRIG): simple prokaryote genome comparisons. BMC Genomics 12:402. 10.1186/1471-2164-12-402, PMID: 21824423 PMC3163573

[ref22] Nagasawa M. Kaku M. Kamachi K. Shibayama K. Arakawa Y. Yamaguchi K.. (2014). Loop-mediated isothermal amplification assay for 16S rRNA methylase genes in gram-negative bacteria. J. Infect. Chemother. 20, 635–638. 10.1016/j.jiac.2014.08.013, PMID: 25179393

[ref23] Nakamatsu E. H. Fujihira E. Ferreira R. C. Balan A. Costa S. O. Ferreira L. C. (2007). Oligopeptide uptake and aminoglycoside resistance in *Escherichia coli* K12. FEMS Microbiol. Lett. 269, 229–233. 10.1111/j.1574-6968.2007.00634.x, PMID: 17250759

[ref24] Nasiri G. Peymani A. Farivar T. N. Hosseini P. (2018). Molecular epidemiology of aminoglycoside resistance in clinical isolates of *Klebsiella pneumoniae* collected from Qazvin and Tehran provinces, Iran. Infect. Genet. Evol. 64, 219–224. 10.1016/j.meegid.2018.06.030, PMID: 29964191

[ref25] Overbeek R. Olson R. Pusch G. D. Olsen G. J. Davis J. J. Disz T.. (2014). The SEED and the rapid annotation of microbial genomes using subsystems technology (RAST). Nucleic Acids Res. 42, D206–D214. 10.1093/nar/gkt1226, PMID: 24293654 PMC3965101

[ref26] Patrick M. Jun H. Robert J. C. Yu Y. Yoon I. K. Robert A. K.. (2012). Complete sequence of a novel 178-kilobase plasmid carrying Bla(NDM-1) in a *Providencia stuartii* strain isolated in Afghanistan. Antimicrob. Agents Chemother. 56, 1673–1679. 10.1128/AAC.05604-11, PMID: 22290972 PMC3318346

[ref27] Pericàs J. M. García-de-la-Mària C. Brunet M. Armero Y. García-González J. Casals G.. (2017). Early in vitro development of daptomycin non-susceptibility in high-level aminoglycoside-resistant *Enterococcus faecalis* predicts the efficacy of the combination of high-dose daptomycin plus ampicillin in an in vivo model of experimental endocarditis. J. Antimicrob. Chemother. 72, 1714–1722. 10.1093/jac/dkx016, PMID: 28204495

[ref28] Poole K. (2004). Efflux-mediated multiresistance in gram-negative bacteria. Clin. Microbiol. Infect. 10, 12–26. 10.1111/j.1469-0691.2004.00763.x, PMID: 14706082

[ref29] Qiong W. Yibo Z. Lizhong H. Jingyong S. Yuxing N. (2009). Plasmid-mediated 16S rRNA methylases in aminoglycoside-resistant *Enterobacteriaceae* isolates in Shanghai, China. Antimicrob. Agents Chemother. 53, 271–272. 10.1128/AAC.00748-08, PMID: 18955532 PMC2612184

[ref30] Sabtcheva S. Saga T. Kantardjiev T. Ivanova M. Ishii Y. Kaku M. (2008). Nosocomial spread of armA-mediated high-level aminoglycoside resistance in *Enterobacteriaceae* isolates producing CTX-M-3 beta-lactamase in a cancer hospital in Bulgaria. J. Chemother. 20, 593–599. 10.1179/joc.2008.20.5.593, PMID: 19028622

[ref31] Siguier P. Perochon J. Lestrade L. Mahillon J. Chandler M. (2006). ISfinder: the reference Centre for bacterial insertion sequences. Nucleic Acids Res. 34, D32–D36. 10.1093/nar/gkj014, PMID: 16381877 PMC1347377

[ref32] Teng X. Jian W. Jianchao Y. Tingyuan Z. Yabo L. Lei X.. (2018). Characterisation of a class 1 integron associated with the formation of quadruple blaGES-5 cassettes from an IncP-1β group plasmid in *Pseudomonas aeruginosa*. Int. J. Antimicrob. Agents 52, 485–491. 10.1016/j.ijantimicag.2018.07.002, PMID: 30012438

[ref33] Wang Y. Shen M. Yang J. Dai M. Chang Y. Zhang C.. (2016). Prevalence of carbapenemases among high-level aminoglycoside-resistant *Acinetobacter baumannii* isolates in a university hospital in China. Exp. Ther. Med. 12, 3642–3652. 10.3892/etm.2016.3828, PMID: 28101158 PMC5228107

[ref34] Xiang-Dang D. De-Xi L. Gong-Zheng H. Yang W. Yan-Hong S. Cong-Ming W.. (2012). Tn1548-associated armA is co-located with qnrB2, aac(6′)-Ib-cr and blaCTX-M-3 on an IncFII plasmid in a *Salmonella enterica subsp. enterica* serovar Paratyphi B strain isolated from chickens in China. J. Antimicrob. Chemother. 67, 246–248. 10.1093/jac/dkr407, PMID: 21965429

[ref35] Ya H. Lu L. Xiaoxia Z. Yu F. Zhiyong Z. (2017). In vitro activity of neomycin, streptomycin, paromomycin and apramycin against carbapenem-resistant *Enterobacteriaceae* clinical strains. Front. Microbiol. 8:2275. 10.3389/fmicb.2017.02275, PMID: 29250040 PMC5715380

[ref36] Yatao G. Yao Z. Zhao Z. Daixi L. Zhanwei W. Jingquan L.. (2018). Complete genomic analysis of a kingdom-crossing *Klebsiella variicola* isolate. Front. Microbiol. 9:2428. 10.3389/fmicb.2018.02428, PMID: 30356723 PMC6189331

[ref37] Yu-Ting D. Zhen-Ling Z. Wei T. Tong Y. Jian-Hua L. (2013). Prevalence and characteristics of rmtB and qepA in *Escherichia coli* isolated from diseased animals in China. Front. Microbiol. 4:198. 10.3389/fmicb.2013.00198, PMID: 23874331 PMC3710952

