# Supplementary material for: High-Level Aminoglycoside Resistance in Human Clinical Klebsiella pneumoniae Complex Isolates and Characteristics of armA-Carrying IncHI5 Plasmids
Source: Front Microbiol. 2021 Apr 7;12:636396. doi: 10.3389/fmicb.2021.636396 (PMC8058188; doi:10.3389/fmicb.2021.636396)
Supplement: Supplementary file 1 [file Table_1.docx]

**TABLE S1**┃Primer sequences used for detection of resistant genes

| Gene | Primer sequence (5’-3’) | Product (bp) | Annealing temperature (℃) | Reference |
| --- | --- | --- | --- | --- |
| *ant(3'')-IIa* | F: GCCGAAGTATCGACTCAAC  R: GCGAGGCCAAGCGATCTTCTTC | 700 | 55 | This study |
| *aadA16* | F: CATCGAGCATCATCTGGGAT  R: ACATTCCGCTCGTCGCCTGC | 501 | 57 | 2 (modified) |
| *aadA5* | F: GATCGAGCGCCATCTGG  R: ACGTTCCGCTCGTCACCCTT | 501 | 57 | 2 (modified) |
| *aac(6')-IIc* | F: CATGCTTGGCTGAACCGCC  R: CCGGCCTTCTCGTAGCA | 395 | 55 | This study |
| *aac(3)-IV* | F: TCGGTCAGCTTCTCAACCTT  R: GATGATCTGCTCTGCCTGTG | 314 | 55 | 1 |
| *aph(3')-Ia* | F: GCGTTGCCAATGATGTTACAG  R: CGAGCATCAAATGAAACTGC | 624 | 55 | 1 |
| *aac(3)-IIg* | F: AACAATTGCGGCGGACCTTTCACG  R: CGCTGCGGATCGGCATTTCGTAGC | 563 | 60 | This study |
| *aac(3)-IId* | F: GGCAATAACGGAGGCGCTTCAAAA  R: TTCCAAGCATCGGCATCTCATACG | 563 | 55 | 1 (modified) |
| *aadA2* | F: CATTGAGCGCCATCTGGAAT  R: ACATTTCGCTCATCGCCGGC | 501 | 57 | 2 |
| *aph(3'')-Ib* | F: GTGGCTTGCCCCGAGGTGATCA  R: CCAAGTCAGAGGGTCCAATC | 612 | 55 | 3 |
| *aph(3')-IIa* | F: GAACAAGATGGATTGCACGC  R: GCTCTTCAGCAATATCACGG | 680 | 55 | This study |
| *aph(4)-Ia* | F: CTGAACTCACCGCGACGTCT  R: TCCACTATCGGCGAGTACTT | 977 | 58 | 4 |
| *aph(6)-Id* | F: ATCGTCAAGGGATTGAAACC  R: GGATCGTAGAACATATTGGC | 509 | 50 | 5 |
| *armA* | F: ATTCTGCCTATCCTAATTGG  R: ACCTATACTTTATCGTCGTC | 315 | 55 | 6 |
| *rmtB* | F: GCTTTCTGCGGGCGATGTAA  R: ATGCAATGCCGCGCTCGTAT | 173 | 55 | 6 |
| *aac(6’)-Ib3* | F: TTGCGATGCTCTATGAGTGGCTA  R: CTCGAATGCCTGGCGTGTTT | 482 | 60 | 1 |

**References**

1.Fernández-Martínez M, Miro E, Ortega A, et al. Molecular identification of aminoglycoside-modifying enzymes in clinical isolates of *Escherichia coli* resistant to amoxicillin/clavulanic acid isolated in Spain. Int J Antimicrob Agents 2015; 46:157-163.

2.Chuanchuen, R., Padungtod, P. Antibiotic resistance genes in Salmonella enterica isolates from poultry and swine. J. Vet. Med. Sci. 2009;70: 1349–55.

3. Elisenda Miró, Federico Grünbaum, Laura Gómez, et al. Characterization of aminoglycoside-modifying enzymes in enterobacteriaceae clinical strains and characterization of the plasmids implicated in their diffusion. Microb Drug Resist, 2013, 19(2):94-99.

4. Lu Nie, Yuemeng Lv, Min Yuan,et al.Genetic basis of high level aminoglycoside resistance in Acinetobacter baumannii from Beijing, China. Acta Pharm Sin B, 2014; 4(4): 295-300

5. Jesús Navas, Marta Fernández-Martínez, Salas C, et al. Susceptibility to Aminoglycosides and Distribution of aph and aac(3)-XI Genes among Corynebacterium striatum Clinical Isolates. PLoS ONE, 2016, 11(12): e0167856.

6. Doi, Y., Arakawa, Y.16S ribosomal RNA methylation: emerging resistance mechanism against aminoglycosides. Clin Infect Dis 2007; 45, 88–94
