# Supplementary material for: High-Level Aminoglycoside Resistance in Human Clinical Klebsiella pneumoniae Complex Isolates and Characteristics of armA-Carrying IncHI5 Plasmids
Source: Front Microbiol. 2021 Apr 7;12:636396. doi: 10.3389/fmicb.2021.636396 (PMC8058188; doi:10.3389/fmicb.2021.636396)
Supplement: Supplementary file 2 [file Table_2.docx]

**TABLE S2**┃Antimicrobial susceptibility(mg/L) and aminoglycoside resistance gene distributions of 40 HLAR strains

| Strain | S | To | Nt | Ak | K | Gm | Aminoglycoside resistance genes |
| --- | --- | --- | --- | --- | --- | --- | --- |
| KP1878 | 4 | >256 | >512 | >1024 | >1024 | >256 | *rmtB* |
| KP1880 | 128 | >256 | >512 | >1024 | >1024 | >256 | *rmtB,aph(6)-Id,aadA16,aph(3'')-Ib,aac(3)-IId, aph(3')-Ia* |
| KP1882 | 1 | >256 | >512 | >1024 | >1024 | >256 | *rmtB* |
| KP1930 | 1 | >256 | >512 | >1024 | >1024 | >256 | *rmtB* |
| KP1934 | 16 | >256 | 32 | 16 | >1024 | 16 | *aph(4)-Ia,aac(3)-IV,aadA2,aph(3')-Ia,aac(6’)-Ib3* |
| KP1942 | 1 | >256 | >512 | >1024 | >1024 | >256 | *rmtB* |
| KP1944 | 1 | >256 | >512 | >1024 | >1024 | >256 | *rmtB* |
| KP1946 | 1 | >256 | >512 | >1024 | >1024 | >256 | *rmtB* |
| KP2073 | 1 | >256 | >512 | >1024 | >1024 | >256 | *rmtB* |
| KP2075 | 1 | >256 | >512 | >1024 | >1024 | >256 | *rmtB* |
| KP2079 | 2 | >256 | >512 | >1024 | >1024 | >256 | *rmtB* |
| KP2097 | 2 | >256 | >512 | >1024 | >1024 | >256 | *rmtB* |
| KP2107 | 2 | >256 | >512 | >1024 | >1024 | >256 | *rmtB* |
| KP2109 | 1 | >256 | >512 | >1024 | >1024 | >256 | *rmtB* |
| KP2125 | 256 | 0.5 | 1 | 1 | >1024 | 1 | *aph(6)-Id,aph(3')Ia* |
| KP2141 | 1 | >256 | >512 | >1024 | >1024 | >256 | *rmtB* |
| KP2151 | 1 | >256 | >512 | >1024 | >1024 | >256 | *rmtB* |
| KP2155 | 64 | >256 | >512 | >1024 | >1024 | >256 | *rmtB, aadA2* |
| KP2159 | 512 | 8 | 8 | 2 | >1024 | 64 | *aac(3)-IId,aph(6)-Id,aadA16,aph(3")-Ib* |
| KP2163 | 2 | >256 | >512 | >1024 | >1024 | >256 | *rmtB* |
| KP2171 | 256 | 8 | 8 | 8 | >1024 | 64 | *aph(6)-Id,aadA16,aph(3')-Ia,aac(6’)-Ib3* |
| KP2717 | 1 | >256 | >512 | >1024 | >1024 | >256 | *rmtB* |
| KP2723 | 2 | 64 | 128 | 16 | 128 | 256 | *aac(6')-IIc,aac(3)-IIg,aac(6’)-Ib3* |
| KP2757 | 1024 | >256 | >512 | >1024 | >1024 | >256 | *armA,aadA2, aph(6)-Id,aac(6’)-Ib3,*  *aac(3)-IId,aph(3'')-Ib,aadA5* |
| KP2779 | 16 | >256 | >512 | >1024 | >1024 | >256 | *rmtB,aph(3')-Ia, aadA2* |
| KP2783 | 16 | >256 | >512 | >1024 | >1024 | >256 | *rmtB,aph(3')-Ia,aadA2* |
| KP2799 | 2 | 8 | 2 | 4 | >1024 | 0.25 | *aph(3’)-Ia* |
| KP2809 | 1 | >256 | >512 | >1024 | >1024 | >256 | *rmtB* |
| KP3018 | 256 | 8 | 4 | 1 | >1024 | 32 | *aac(3)-IId,aph(3')-Ia,aph(6)-Id,aadA16* |
| KP3036 | 4 | 16 | 16 | 2 | >1024 | 64 | *aadA2,aadA16,aac(3)-IId* |
| KP3048 | 16 | >256 | >512 | >1024 | >1024 | >256 | *rmtB, aadA2* |
| KP3050 | 8 | >256 | >512 | >1024 | >1024 | >256 | *rmtB, aadA2* |
| KP3052 | 256 | 16 | 16 | 4 | >1024 | 32 | *aph(6)-Id, aadA16, aac(3)-IId, aph(3'')-Ib, aph(3')-Ia, aac(6’)-Ib3* |
| KP3062 | 8 | >256 | >512 | >1024 | >1024 | >256 | *rmtB, aadA2* |
| KP3064 | 8 | >256 | >512 | >1024 | >1024 | >256 | *rmtB, aadA2* |
| KP3078 | 256 | 16 | 32 | 4 | >1024 | 64 | *aph(6)-Id, aadA16, aac(3)-IId, aph(3'')-Ib* |
| KP3088 | 256 | 16 | 32 | 4 | >1024 | 64 | *aph(3'')-Ib, aadA16,aph(6)-Id ,aac(3)-IId* |
| KP3092 | 16 | >256 | >512 | >1024 | >1024 | >256 | *rmtB, aadA2, aadA5* |
| KP3113 | 16 | >256 | >512 | >1024 | >1024 | >256 | *rmtB, aadA2* |
| KP4042 | 2 | >256 | >512 | >1024 | >1024 | >256 | *rmtB,aph(3')-IIa* |

S, streptomycin;To, tobramycin; Nt, netilmicin; Ak, amikacin;K, kanamycin; Gm, gentamicin
