# Supplementary material for: High-Level Aminoglycoside Resistance in Human Clinical Klebsiella pneumoniae Complex Isolates and Characteristics of armA-Carrying IncHI5 Plasmids
Source: Front Microbiol. 2021 Apr 7;12:636396. doi: 10.3389/fmicb.2021.636396 (PMC8058188; doi:10.3389/fmicb.2021.636396)
Supplement: Supplementary file 3 [file Table_3.docx]

**TABLE S3**┃Antimicrobial susceptibility (mg/L) of 40 HLAR strains

| Strain | CZ | CX | CAZ | AZM | CIP | P/T | IPM | DOX | CL | FOS | FEP |
| --- | --- | --- | --- | --- | --- | --- | --- | --- | --- | --- | --- |
| KP1878 | >128 | >128 | 64 | >128 | >16 | 512/4 | >16 | 64 | >128 | 512 | >64 |
| KP1880 | >128 | 128 | 64 | >128 | >16 | 512/4 | >16 | 64 | >128 | 512 | >64 |
| KP1882 | >128 | 128 | 32 | >128 | >16 | 512/4 | >16 | 64 | >128 | >1024 | >64 |
| KP1930 | >128 | 64 | 32 | >128 | >16 | 512/4 | >16 | 8 | >128 | 512 | >64 |
| KP1934 | >128 | >128 | 16 | 4 | >16 | 128/4 | 16 | 4 | >128 | 256 | 32 |
| KP1942 | >128 | 64 | 32 | >128 | >16 | 256/4 | >16 | 8 | >128 | 512 | >64 |
| KP1944 | >128 | 64 | 64 | >128 | >16 | 512/4 | >16 | 4 | >128 | 512 | >64 |
| KP1946 | >128 | 128 | 32 | >128 | >16 | 256/4 | >16 | 4 | >128 | 512 | >64 |
| KP2073 | >128 | 128 | 32 | >128 | >16 | 256/4 | >16 | 4 | >128 | 512 | >64 |
| KP2075 | >128 | 128 | 16 | >128 | >16 | 256/4 | >16 | 4 | >128 | 512 | >64 |
| KP2079 | >128 | 8 | 16 | 64 | 2 | 4/4 | 0.5 | 32 | 64 | >1024 | 32 |
| KP2097 | >128 | 128 | 32 | >128 | >16 | 256/4 | >16 | 8 | >128 | 512 | >64 |
| KP2107 | >128 | 128 | 32 | >128 | >16 | 128/4 | >16 | 8 | >128 | 1024 | >64 |
| KP2109 | >128 | >128 | 32 | >128 | >16 | 256/4 | >16 | 8 | >128 | 1024 | >64 |
| KP2125 | >128 | 32 | 8 | >128 | 0.5 | 4/4 | 1 | 64 | 128 | 32 | 64 |
| KP2141 | >128 | 128 | >64 | >128 | >16 | 512/4 | >16 | 8 | >128 | 512 | >64 |
| KP2151 | >128 | 128 | 32 | >128 | >16 | 512/4 | >16 | 8 | >128 | 512 | >64 |
| KP2155 | >128 | >128 | 32 | >128 | >16 | 256/4 | >16 | 16 | 16 | >1024 | >64 |
| KP2159 | >128 | 16 | 32 | 128 | >16 | 256/4 | 16 | >64 | 128 | 512 | 16 |
| KP2163 | >128 | 64 | 8 | >128 | >16 | 4/4 | 1 | 8 | >128 | 512 | 64 |
| KP2171 | >128 | 16 | 16 | 128 | >16 | 256/4 | 4 | >64 | >128 | 64 | 32 |
| KP2717 | >128 | 2 | 2 | 32 | >16 | 2/4 | 0.5 | 64 | >128 | 64 | 4 |
| KP2723 | >128 | >128 | >64 | >128 | 2 | 16/4 | 0.5 | >64 | >128 | >1024 | 1 |
| KP2757 | >128 | >128 | 8 | 32 | 2 | 8/4 | 2 | 32 | 16 | 1024 | 64 |
| KP2779 | >128 | >128 | >64 | >128 | >16 | 512/4 | >16 | 16 | >128 | >1024 | >64 |
| KP2783 | >128 | >128 | 64 | >128 | >16 | >512/4 | >16 | 16 | >128 | >1024 | >64 |
| KP2799 | >128 | >128 | 16 | 4 | >16 | 8/4 | 1 | 4 | 128 | 256 | 2 |
| KP2809 | >128 | 128 | 32 | >128 | >16 | 512/4 | >16 | 8 | 16 | 256 | >64 |
| KP3018 | >128 | 8 | 8 | 32 | >16 | 8/4 | 0.5 | 16 | 16 | 32 | 16 |
| KP3036 | >128 | 8 | 64 | 64 | >16 | 4/4 | 1 | 64 | 128 | 64 | 32 |
| KP3048 | >128 | >128 | 64 | >128 | >16 | 512/4 | >16 | 16 | >128 | 256 | >64 |
| KP3050 | >128 | 128 | 32 | >128 | >16 | 512/4 | >16 | 16 | 32 | >1024 | >64 |
| KP3052 | >128 | 8 | 4 | 32 | 8 | 4/4 | 1 | 64 | 128 | 64 | 32 |
| KP3062 | >128 | >128 | 64 | >128 | >16 | 512/4 | >16 | 8 | 32 | >1024 | >64 |
| KP3064 | >128 | >128 | 64 | >128 | >16 | 512/4 | >16 | 16 | 16 | >1024 | >64 |
| KP3078 | >128 | 4 | 4 | 32 | 8 | 4/4 | 1 | 64 | >128 | 32 | 32 |
| KP3088 | >128 | 8 | 8 | 32 | 8 | 4/4 | 1 | 64 | >128 | 32 | 32 |
| KP3092 | >128 | >128 | 64 | >128 | >16 | 512/4 | >16 | 32 | 16 | >1024 | >64 |
| KP3113 | >128 | 4 | 32 | >128 | >16 | 512/4 | >16 | 64 | 64 | >1024 | >64 |
| KP4042 | 2 | 2 | 0.25 | 1 | 0.03 | 2/4 | 1 | 8 | 8 | 128 | 0.25 |

Cefazolin, CZ; Cefoxitin, CX; Ceftazidime, CAZ; Aztreonam, AZM; Ciprofloxacin, CIP; Piperacillin tazobactam, P/T；Imipenem, IPM; Doxycycline, DOX；Chloramphenicol, CL; Fosfomycin, FOS; Cefepime, FEP;
