# Supplementary material for: High-Level Aminoglycoside Resistance in Human Clinical Klebsiella pneumoniae Complex Isolates and Characteristics of armA-Carrying IncHI5 Plasmids
Source: Front Microbiol. 2021 Apr 7;12:636396. doi: 10.3389/fmicb.2021.636396 (PMC8058188; doi:10.3389/fmicb.2021.636396)
Supplement: Supplementary file 4 [file Table_4.docx]

**TABLE S4**┃Plasmid sequences used for phylogenetic and comparative genomic analyses in this study

| Plasmid | Acc. number | Species | Origin | ARI-A^a^ ARI-B^a^ | |
| --- | --- | --- | --- | --- | --- |
| p19051-IMP | MF344565 | *K. pneumonia* | Homo sapiens | *armA* | *aph(3’’)-Ib, aph(6)-Id, aac(6’)-Ib3, aadA2, armA, aac(3)-IId* |
| p2757-346 | CP060810 | *K. variicola* | Homo sapiens | *armA, aadA5* | *aph(3’’)-Ib, aph(6)-Id, aac(6’)-Ib3, aadA2, armA, aac(3)-IId* |
| p11219-IMP | MF344561 | *K. pneumoniae* | Homo sapiens | *armA* | *aph(3’’)-Ib, aph(6)-Id, aac(6’)-Ib3, aadA2, armA* |
| p13450-1 | CP026014 | *K. variicola* | Homo sapiens | *armA, aaA2, aac(6’)-Ib3* | *aph(3’’)-Ib, aph(6)-Id, armA, aac(3)-IId* |
| pKOX_R1 | CP003684 | *K. michiganensis* | Homo sapiens | *armA, aaA2, aac(6’)-Ib3* | *aph(3’’)-Ib, aph(6)-Id, aadA5, armA, aac(3)-IId* |
| p12208-IMP | MF344562 | *K. pneumoniae* | Homo sapiens | *armA, aac(3)-IId* | *armA* |
| pSIM-1-BJ01 | MH681289 | *K. pneumoniae* | Homo sapiens | *armA, aadA5* | *aadA17, aph(3’’)-Ia* |
| pWLK-238550 | CP038277 | *Raoultella ornithinolytica* | Sediment | *armA, aadA5* | *aac(6’)-Ib-cr, armA* |

^a^Aminoglycosides resistance genes shown only.
